# Wellness: Combating Burnout and Its Consequences in Emergency Medicine

**DOI:** 10.5811/westjem.2020.1.40971

**Published:** 2020-04-13

**Authors:** Christine R. Stehman, Ryan L. Clark, Andrea Purpura, Adam R. Kellogg

**Affiliations:** * Indiana University School of Medicine, Department of Emergency Medicine, Indianapolis, Indiana; † University of Massachusetts Medical School – Baystate Health, Department of Emergency Medicine, Springfield, Massachusetts

## Abstract

Medicine recognizes burnout as a threat to quality patient care and physician quality of life. This issue exists throughout medicine but is notably prevalent in emergency medicine (EM). Because the concept of “wellness” lacks a clear definition, attempts at ameliorating burnout that focus on achieving wellness make success difficult to achieve and measure. Recent work within the wellness literature suggests that the end goal should be to achieve a culture of wellness by addressing all aspects of the physician’s environment. A review of the available literature on burnout and wellness interventions in all medical specialties reveals that interventions focusing on individual physicians have varying levels of success. Efforts to compare these interventions are hampered by a lack of consistent endpoints. Studies with consistent endpoints do not demonstrate clear benefits of achieving them because improving scores on various scales may not equate to improvement in quality of care or physician quality of life. Successful interventions have uncertain, long-term effects. Outside of EM, the most successful interventions focus on changes to systems rather than to individual physicians. Within EM, the number of well-structured interventions that have been studied is limited. Future work to achieve the desired culture of wellness within EM requires establishment of a consistent endpoint that serves as a surrogate for clinical significance, addressing contributors to burnout at all levels, and integrating successful interventions into the fabric of EM.

## INTRODUCTION

In part one of this two-part series, we explored burnout – its definitions, causes, and consequences – with a specific focus on burnout in emergency medicine (EM).^1^ To begin to address burnout, we must understand the end goal, which for many is the opposite of burnout, the nebulous construct of wellness.

The National Wellness Institute (NWI) defines wellness as “an active process through which people become aware of, and make choices toward, a more successful existence.”^2^ Developed in 1976, this is probably the most frequently used wellness paradigm; however, wellness models involve all aspects of a person’s life, their environment, and surrounding community (Appendix 1). Recent research confirms that the domains used by the NWI offer guidance on improving and promoting *personal* wellness.^3^–^5^ But personal wellness, the sole focus of the NWI and many wellness models, is just one part of a physician’s life. As evidenced by the contributors to burnout described in part one of this series, including clinical pressures, shift work, and electronic health records (EHR), physicians operate in an organizational structure that may either enhance or degrade their personal and professional wellness.^1^

Multiple experts define wellness in medicine similarly to the World Health Organization (WHO) definition of health (“a state of complete physical, mental and social well-being and not merely the absence of disease or infirmity”).^6^ Bart et al, in their systematic review of wellness literature in clinical medicine, showed that the current working definition of wellness revolves around the WHO definition and the 1998 Wheel of Wellness.^7^–^8^ However, these authors and others agree that the term wellness in clinical medicine “lacks a singular definition.”^7^,^9^–^10^

The ideal model for physician wellness should include both individual factors and the organizational structure and environment in which physicians work. Three such models exist: Stanford’s WellMD Initiative; the Mayo Clinic’s Engagement Model; and the National Academy of Medicine (NAM) conceptual model of Clinician Well-being and Resilience (Table 1).^11^–^14^ These models describe a complex interplay of personal and organizational factors, suggesting that interventions to combat burnout must address both factors surrounding the physicians *and* perceived physician shortcomings.

Developers of burnout interventions must be able to determine the success or failure of the intervention. Eckleberry-Hunt et al suggest measuring wellness rather than burnout to avoid the negativity associated with the term burnout and promote the positive aspects of achieving wellness.^10^ While many wellness scales have been used in the physician population, these measures have not been evaluated as extensively as burnout scales such as the Maslach Burnout Inventory (MBI). Few show associations between improving scores and improving clinical outcomes (eg, fewer perceived medical errors), making it difficult to determine which interventions have clinical significance.^9^,^15^

We examine what has been done to address physician well being in general, as well as specifically in EM, and highlight the interventions showing a clinical improvement associated with their measurements. We then describe what the future interventions tailored to EM should be, as we endeavor to improve the culture, environment and overall well being of EM as a whole.

## METHODS

### Keywords

For this study we chose to combine words from three different categories to find interventions aimed at improving wellness/combating burnout: 1) words reflecting what was done – intervention, therapy, treatment, solutions; 2) words describing the problem being addressed – burnout, wellness, well-being, resilience, compassion fatigue, as well as specific contributors to burnout/lack of well being in EM (electronic health records [EHR], sleep, fatigue, shift work, shift work sleep disorder, second victim syndrome, litigation stress, financial stress, debt); and 3) relevant population keywords – physicians, medical students, residents. The term “emergency medicine” was added to find EM-specific literature.

### Search

We searched all combinations of the three categories of keywords (“what was done” + “problem” + “population”) from 1974 to the present in both Ovid Medline and PubMed. We also searched EM and critical care blogs for relevant articles/posts.

### Article Inclusion Criteria

We categorized all search results into primary studies, meta-analyses/systematic reviews, commentary/opinion pieces, and general review articles. Primary research studies as well as the meta-analyses/systematic reviews, inclusive of their relevant references, provided the database of supporting information for the composition of this review. Additionally, we attempted to identify the primary literature for all Internet-based resources.

## RESULTS

Interventions aimed at promoting well being and ameliorating burnout abound in all specialties, mostly focusing on person-directed interventions such as teaching mindfulness and improving resilience. Table 2 details the systematic reviews/meta-analyses of the extensive number of articles available. All interventions included in these articles focused on changes in the scores on various burnout/depression scales, usually the MBI. In general, the systematic reviews/meta-analyses confirm previous research: interventions appear to make small but statistically significant reductions in emotional exhaustion, burnout, and stress. A few of these articles specifically conclude that organization-level interventions impact physician scores as much as, if not more than, the person-based interventions and should be included in any intervention program.^16^,^19^–^20^

Fewer intervention studies have been published in EM (Table 3). These interventions focus on changes in scores on scales such as the MBI and have mixed results. Because the literature is so sparse in this area, we also included interventions in any discipline focusing on the specific contributors to EM burnout described in part one of this series (the EHR, sleep deprivation/fatigue/shift work, metrics/clinical pressures, second victim syndrome, litigation stress, and financial stressors).^1^ Table 4 details the results of these searches. Again, there are limited targeted interventions applicable to EM.

## DISCUSSION

Organizations have tried multiple types of interventions to improve wellness and combat burnout, often focusing on how individual physicians can improve their circumstances. These interventions serve to improve scores on various burnout and well-being scales, and, in some cases, showed improvement in quality of care measures (Tables 2 and 3).

For example, Braun et al. found that mindfulness-based interventions trended towards improvements in self-reported patient care, patient-centered care, patient satisfaction, and patient symptomatology.^25^ Unfortunately, it is hard to extrapolate how these interventions might affect emergency physicians (EPs) since all those interventions centered on mental health professionals. One mindfulness-based intervention has been tried on students rotating through the emergency department (ED). Chung et al found self-reported, improved behaviors and attitudes in the students who experienced the curriculum.^28^ This intervention, like many of the non-EM studies, was short term, which means it is uncertain how long, if at all, the results last.

The improvements in burnout and wellness scores seen in these and other studies show that this focus on person-based interventions remains important. Self-care and individual mindsets influence how differently people are affected by their environment, leading to variable development of burnout among physicians working under the same circumstances. Unfortunately (but possibly correctly), physicians tend to infer that the default to person-based interventions places the blame for burnout and the associated consequences solely on the physician.^47^–^48^ In addition, years of these person-based interventions have demonstrated little to no improvement in the overall burnout levels of physicians,^49^–^50^ indicating that person-based interventions are necessary but insufficient to fully address the problem.

The perception of many physicians, as supported by studies discussed in part one of this series, is that the key contributors to burnout lie outside of individual physician control.^1^,^13^,^50^–^55^ The developers of the NAM Conceptual Model of Wellness highlight the influence that organizational, systemic, environmental, and societal issues have on burnout and suggest that attention must be paid to these details to truly have a long-term effect on this issue.^14^

Some organizations have started to implement interventions aimed at fixing such system and organizational issues. While data from these interventions is limited, they seem to have a more profound improvement on burnout scores than person-based interventions. Shanafelt et al details the improvements seen across the board with the implementation of such changes.^13^ While none of these interventions took place explicitly in EM, they support the need for organizations and society as a whole to recognize their role in contributing to physician burnout in all specialties. In addition, organizations and societies will need to do more than be supportive of physician well being if their goal is to alleviate the problem of burnout.

When it comes to burnout and well being, EM is unique in a number of ways. EPS work fewer hours than physicians in most other specialties (a primary contributor to burnout in general) and their job satisfaction is frequently high despite high levels of burnout.^50^,^56^ This uniqueness may limit how successful interventions in other specialties will apply to EPs. Because of the need to account for the seemingly contradictory facets of EM when considering interventions, studies need to be done with EPs. This has rarely been done. The interventions studied in EM tend to focus on person-based interventions and have had mixed results: Some show improvement in the varied endpoints chosen while others do not.

Other studies of EPs focus on interventions affecting the contributors to burnout in EM. These interventions examined a variety of endpoints with some subsequent improvement, although it is hard to understand how these changes affect burnout and wellness as changes to scales in these domains were rarely examined (Table 4).

There are two issues with all the studies regarding wellness and burnout interventions, both within EM and other specialties. First, each study involves an intervention lasting no longer than a year with limited post-intervention follow-up. The lack of long-term follow-up means that there is uncertainty about whether the changes, positive or negative, persist after the end of the intervention. If there is no lasting change from the intervention, then the intervention may end up being harmful as it created a false sense of improvement. The second, and probably more concerning, difficulty with these studies is that few interventions actually take the results a step further and evaluate more than just scores on various wellness and burnout scales. While research shows that physicians suffering from burnout provide worse patient care in multiple ways, no intervention studies have examined whether improvement in those scales after the intervention is associated with improved quality of care.^57^–^60^

Some of the mindfulness-based interventions studies reviewed by Braun et al suggest that these interventions improve patient care; however, they do not have associated changes in burnout or wellness scores for other studies to compare with.^25^ Robinson et a. evaluated the effect of EHR training on a large number of physicians and found fewer medical errors.^40^ The authors drew the conclusion that improved interface with the EHR may improve burnout by decreasing the time spent with the computer rather than the patient, but they also did not check before and after scores on any of the burnout or well-being scales.^40^ While statistically significant improvements in burnout and well-being scales are considered to be appropriate surrogates for the problem facing physicians, no one has yet shown actual clinical significance in these changes.

The development of the NAM Conceptual Model of Wellness, as well as the apparent successes at both Mayo and Stanford based on organizational changes, reinforces that improving physician well being/combating burnout will require the establishment of a culture of wellness.^11^–^14^ A culture of wellness, defined by Stanford as “a work environment with a set of normative values, attitudes and behaviors that promote self-care, personal and professional growth, and compassion for colleagues, patients and self,” is probably a distant goal for EM.^11^ Before we as EPs can establish what values, attitudes, and behaviors would serve us best, we must determine a number of other things in the realm of physician well-being.

### Measuring the Problem and Outcomes

Lall et al describe the numerous burnout and well-being scales previously used to assess physicians, and that are available for use in evaluating interventions.^9^,^61^ They also describe the downsides of these scales and allude to the bigger problem: We do not yet know which is the most appropriate scale for use in EM.^9^,^61^ Should we be highlighting the issue by using a burnout scale? While the most widely used “default” burnout scale is simple to use, there are a number of issues with how the MBI and other burnout measures are being used, as described by Eckleberry-Hunt et al.^62^ In addition, knowing that a study is evaluating burnout risks may alter the participants’ responses, as this term (along with wellness) has been used so frequently that many people are “sick of it.”^62^–^63^

Should we instead be seeking the “bright spots” (people in high-risk environments who are thriving) by using a well-being scale?^64^ Given how pervasive burnout seems to be in EM (ranging from 38% to as high as 74%), seeking the “bright spots” within the specialty would allow investigators to focus on the positive.^65^–^66^ In addition, finding out what “bright spots” do differently may lead EPs to possible interventions that have not yet been considered.

### Clinical Significance

Investigators need to determine whether changes in potential scales actually tie to clinically significant outcomes. Everything being done to treat the problem of burnout is because both physicians and patients are suffering.^49^,^53^,^57^–^60^,^67^–^69^ It is not enough that interventions change burnout or wellness scores. Ultimately, they must improve physician quality of life and the quality of patient care provided; otherwise, investigators are either using the wrong surrogate for the problem or evaluating an ineffective intervention. This is a confounding variable that needs to be figured out before implementing large-scale interventions.

### Interventions

After investigators establish a common measurement to use, work needs to be done on the interventions themselves. Person-based interventions likely do have a place in the treatment of burnout as these interventions have been shown to result in improvement in measurement scores and may result in real-life improvements as well. However, physicians are wary of them given that these interventions seem to place blame on the physicians for being burned out.^48^ In order to continue to involve successful person-based interventions in future programs, experts will need to overcome this wariness. This will likely require demonstrating that these interventions are only part of a broader intervention. Investigators will need to encourage physician engagement, truly involving physicians in the development of these programs.

Finally, the focus of future, person-based interventions will likely need to shift. Physicians of all levels, as early as medical school, suffer from burnout.^70^ Thus, it is likely that something predisposes us to burnout, possibly the same characteristics that push us to continue operating in toxic environments for the good of our patients. Simply improving mindfulness, changing the way physicians eat, sleep or workout, or focusing on resilience will likely have little long-term impact. However, a shift to physicians living their values and learning to set boundaries so they help change their environments will likely have a greater long-term impact on overall physician wellness, while encouraging physicians to engage more in the conversation.

In addition, these person-based interventions often ask physicians to cope with unsustainable work conditions, rather than fixing the conditions in which they work. As discussed in part one of this series, many organizational, environmental, and societal factors contribute to the development of burnout and the associated decreased quality of care.^1^ More recent non-EM interventions have shown success by incorporating organizational and environmental changes to address these conditions.^13^ Future work in EM will involve determining which of these factors relevant to EM are amenable to intervention, how to address these factors in the face of the inevitable pushback from those who have benefited from the status quo, and how to successfully combine changes to these factors with interventions aimed at person-based issues. This combination is the key to creating interventions that truly create a culture of wellness in EM.

One particular organizational and societal issue is felt keenly in EM. In the 45 years of our specialty, there has been a transition away from physician autonomy and the primacy of the physician-patient relationship. Now, outside entities dictate how physicians practice medicine with the focus on making money rather than being motivated by patient care.^71^ While society may need a less-patriarchal physician role in medical decision-making, physicians have trained for a long time to be able to make decisions for the good of their patients; so these decisions should not be made by those who are not trained in medicine. In EM, this lack of autonomy comes in the form of patient satisfaction scores and clinical metrics that check bureaucratic boxes rather than affecting patient care.^56^ Interventions that address this lack of autonomy are likely to engage physicians to a greater extent than those that ignore this issue.

Intervention developers will likely need to look outside of medicine for inspiration to address one unique aspect of our job. While all physicians have patients that could potentially “haunt” them, few other specialties see people on the worst days of their lives multiple times a day. Each day, EPs experience secondary trauma: EPs are not in the car crash, but they see the results and experience the heartbreak along with the family; EPs see the devastation caused by drugs and alcohol as well as the side effects of homelessness, rising drug costs, lack of transportation, illiteracy, and more. EPs experience all of these things while being relatively helpless to cause positive change. In this aspect of their job, EPs are more akin to law enforcement and deployed military. How should EM address the daily secondary trauma from seeing the worst days of people’s lives as well as barriers to care for which EPs sometimes can do nothing?

Also similar to law enforcement professionals, for an EP, achieving true downtime is difficult. Each EP notices situations that could be potentially hazardous and is ready to act in the event he or she is needed. EPs are resilient but their job requires at least one step beyond normal resilience. Some areas of law enforcement have found success in remediating this secondary trauma, the lack of being able to truly be “off” and its side effects which are similar to the burnout and depression seen in EPs (R. Nayi Partridge, Director of Resilience Training and Development at The Partridge Group, email and phone communication, June 2018). EM may find that incorporating variations of successful law-enforcement interventions could be beneficial in addressing these issues.

Finally, previously studied interventions have all been short term both in regard to the length of the intervention and the duration of follow-up after the intervention ends. Future studies need to look at longer duration interventions as well as longer follow-up to ensure that results are sustained. Eventually, the interventions that succeed will hopefully become part of the way EM functions, as the best would represent development of the culture of wellness that is the goal.

## CONCLUSION

Interventions intended to combat burnout and improve wellness in physicians are difficult to interpret for a number of reasons, including short-term duration of interventions and follow-up, variable outcomes, and lack of proven clinical significance. While non-EM initiatives appear numerous, few EM initiatives have been tried. Those interventions showing promise involve changes at both the individual physician level and the organizational and environmental levels. This both shifts the “blame” for burnout and its consequences off the individual physician and acknowledges that burnout is a symptom of a culture problem in medicine, a culture that expects everything of the physician while giving little to nothing back.

Given that these issues seem to stem from the current problem culture of medicine, the ultimate goal of any wellness initiative needs to be a shift in that culture. Organizations seeking to improve physician wellness/combat physician burnout should aim to create a culture of wellness. Creating this culture means addressing every physician’s whole environment: the physicians themselves, the organizations they practice in, and the societal issues they face.

Successful interventions will work to create a culture of wellness and will become part of that culture. However, that goal is distant for all of medicine. Decisions regarding which scale to use to measure success, how to decide whether intervention results are clinically significant, and an acceptable duration to achieve sustained results need to be made. The specialty of emergency medicine can base its interventions on successful non-EM medicine interventions while integrating specific factors that address the unique aspects of the EP’s job, including secondary trauma.

After addressing all of these issues regarding the implementation and evaluation of wellness interventions, physicians will write a cohesive narrative of standards to meet, changes to pursue, and how to change course as needed. This narrative will weave together multiple interventions to read as a sustained, ongoing culture of wellness in EM.

## Supplementary Information



## Figures and Tables

**Table 1:** Models of wellness most applicable to emergency medicine.

Model	Definition of Well-being	Components
Stanford Wellness Framework^11^–^12^	Physician wellness = Professional fulfillment (experience happiness or meaningfulness, self-worth, self-efficacy, and satisfaction at work).	Culture of Wellness: behaviors, attitudes and values that promote self-care and growth (organizational responsibility). Efficiency of Practice: value of clinical practice/(time and energy spent); organizational responsibility. Personal Resilience: personal skills, behaviors and attitudes that contribute to personal well-being (personal obligation).
Mayo Clinic Engagement Model^13^	Defines the opposite of burnout as engagement (vigor, dedication and absorption in work)	Workload and Job Demands: eg, specialty, team structure, compensation, and all metrics. Efficiency and Resources: eg, personal, team and institutional efficiency, personal organization and delegation skills, EHR. Meaning in work: eg, opportunities for advancement, organizational culture, personal values, physician-patient relationship. Organizational Culture and Values: eg, physician’s personal and professional values; organization’s mission, norms, culture and values. Control and Flexibility: eg, physician personality/intentionality and organization’s degree of flexibility on a number of issues. Social Support and Community at Work: eg, physician’s relationship building skills, team structure, organizational collegiality, and promotion of community. Work-life Integration: eg, physician values and personal characteristics, organizational expectations, and requirements for call and cross-coverage.
National Academy of Medicine Conceptual Model^14^	Clinician well being is a multidisciplinary issue that requires a systems-thinking approach to address fully.	Health Care Role: eg, stage in career, patient population, all responsibilities, and alignment of authority and responsibility. Personal Factors: eg, values, personality traits, social support, and physical/mental/spiritual health. Skills and Abilities: eg, teamwork, resilience, coping skills, empathy and leadership skills. Socio-cultural Factors: eg, societal expectations of physicians, political and economic climate, mental health stigmatization, social determinants of health, culture of safety, implicit and explicit biases. Regulatory, Business, and Payer Environment: eg, compensation, documentation requirements, licensing, litigation risk, insurance company policies. Organizational Factors: eg, organizational culture, mission, leadership and values; bureaucracy, diversity and inclusion, level of organizational support, professional development. Learning and Practice Environment: eg, autonomy, relationships, mentorship, EHR, learning, practice setting and environment.

**Table 2:** Systematic reviews of general physician interventions for burnout and wellness.

Author	Year	Number of studies included	Population	Scales/Endpoints	Determination/Conclusion
Wiederhold^16^	2018	13	Physicians	MBI	Develop interventions at the personal and institutional levels.
Busireddy^17^	2017	19	Residents	MBI	ACGME work hour limits decreases emotional exhaustion and burnout.
Clough^18^	2017	23	Physicians	MBI and a variety of stress scales	Occupational stress and burnout helped by cognitive and behavioral interventions.
Panagiotti^19^	2017	19	Physicians	MBI	Small but significant decreases in emotional exhaustion; with larger effect from organizational interventions.
West^20^	2016	42	Physicians	MBI	Individualized and organizational interventions decreased burnout.
Burton^21^	2016	9	Health care professionals (5 studies included physicians)	Variety of measures of stress	Mindfulness-based Interventions decrease stress.
Williams^22^	2014	19	Medical students and residents	Multiple measures of burnout, depression, and suicide rates	Varied results.
Regehr^23^	2014	12	Medical students and Physicians	Variety of burnout, stress, and anxiety scales	Cognitive, behavioral, and mindfulness-based approaches reduce stress.
Awa^24^	2010	25	Health care providers (one physicians only)	Burnout measures, primarily MBI	Greatest and most lasting reductions in burnout were associated with combination of person- and organization-directed interventions.
Braun^25^	2018	26	Health care providers (one physician only)	Various patient care related outcomes such as patient care, patient satisfaction and safety	“There is great potential for [Mindfulness-based interventions] to improve [healthcare provider] functioning and therefore patient care.”

*MBI*, Maslach Burnout Inventory; *ACGME*, Accreditation Council for Graduate Medical Education.

**Table 3:** Burnout and wellness interventions in emergency medicine.

Author	Year	Description of Intervention	Scale used, if any	Results (positive, negative, change in scale)
Hart^26^	2019	Corporate wellness program	MBI	Didn’t like the intervention, worsened burnout
Braganza^27^	2018	Mindfulness workshop with ongoing activities	K10-Psychological distress; MBI	K-10 score decreased significantly; no change in MBI
Chung^28^	2018	Mindfulness curriculum medical students on EM rotation	Behaviors and attitudes (self-reported)	Improved
Schrager^29^	2017	Wearable physical activity trackers	Days per week of physical activity	Increase only among those with low pre-intervention levels
Williamson^30^	2017	Implemented a wellness curriculum at 5 residencies, including lectures, individual activities, and resources	None (descriptive)	Implementation was feasible
Mache^31^	2016	Mental health promotion program for junior Emergency Physicians	Variety of measures including MBI and Perceived Stress Questionnaire	Decreased perceived stress and emotional exhaustion
Gorgas^32^	2015	Emotional Intelligence training for EM residents	Hay 360 Emotional Competency Inventory	Increased EI scores

*MBI*, Maslach Burnout Inventory; *EM*, emergency medicine; *EI*, emotional intelligence.

**Table 4:** Interventions for known contributors to burnout in emergency medicine.

Author	Year	Area of Intervention	Discipline	Description of Intervention	Scale used, if any	Results (positive, negative change in scale)
Walker^33^	2019	EHR	EM	Scribe program implementation in 5 EDs	Patients per hour	Increased physicians’ productivity.
Chung^34^	2018	SVS	EM	Educator toolkit for addressing SVS via mindfulness	None	None
Smith^35^	2016	Litigation	EM	Effect of adding empathetic statements to patient encounters on likelihood to consider litigation	Likelihood of suing a doctor for a misdiagnosis on VAS	Decreased likelihood of suing.
Smith-Coggins^36^	2006	Sleep/Fatigue	EM	Effect of nap at 3 AM on task performance and alertness	Variety of task completion, fatigue, and memory scales; driving simulation	Overall improvement, except brief decrease in memory immediately after the nap.
Croskerry^37^	2002	Sleep/Fatigue/Shift work	EM	Implemented Casino Nights (night shift ending at 0400)	Preference compared to standard night shift and amount of sleep	Casino shift preferred; increased sleep.
Shanafelt^13^	2017	General	General	Collection of organizational interventions implemented by the Mayo Clinic	MBI	7% decrease in burnout.
West^38^	2014	General	Internal Medicine	RCT of effect of a physician facilitated small group curriculum on well-being	MBI and sub-scales	Decreased rates of depersonalization, emotional exhaustion, and overall burnout.
Contratto^39^	2017	EHR	General IM (7 physicians)	Clerical support personnel for EPOE	Attitudes, satisfaction with EHR, productivity	Felt more supported, less fatigued.
Robinson^40^	2018	EHR	3500 physicians	EHR trainings	mixed -methods	Improved documentation, fewer medical errors, increased chart efficiency.
Liira^41^	2014	Sleep loss	Night shift workers	Systematic review of pharmacologic interventions to improve sleep and alertness on shift	Sleep length; the Karolinka Sleepiness Scale	Melatonin increases sleep length; Modafinil and Armodafinil improve alertness.
Linzer^42^	2015	Clinical Pressures	Primary Care	Diverse interventions in communication, workflow changes, and targeted quality improvement projects that included clinician input	5 item burnout scale; satisfaction; intention to leave in 2 years	Decreased burnout and improved satisfaction but no change in intention to leave.
Ey^43^	2016	SVS	General	Comprehensive wellness and suicide prevention program in a health system	Utilization by residents and faculty; satisfaction of trainees and program directors	Progressively increased utilization. High levels of satisfaction.
Miller^44^	2019	SVS	Included 15 articles on interventions	Systematic review of SVS that included mindfulness interventions	Variety of stress and burnout scales	Improvement
Scott^45^	2010	SVS	General	Creation of a systemwide rapid response team for SVS with a multi-tiered deployment model	None	Feasible
Durand^46^	2015	Litigation stress	General	Systematic review of SDM on likelihood of malpractice claims	Varied by study	Some studies showed evidence for benefit with SDM, though provider preference was increased testing

*EHR*, electronic health records; *EM*, emergency medicine; *ED*, emergency department; *SVS*, second victim syndrome; *VAS*, visual analog scale; *MBI*, Maslach Burnout Inventory; *RCT*, Randomized Controlled Trial; *IM*, internal medicine; *EPOE*, electronic provider order entry.

*SVS*, second victim syndrome; *VAS*, visual analog scale; *SDM*, shared decision-making.
