# Supplementary material for: Wellness: Combating Burnout and Its Consequences in Emergency Medicine
Source: West J Emerg Med. 2020 Apr 13;21(3):555–65. doi: 10.5811/westjem.2020.1.40971 (PMC7234720; doi:10.5811/westjem.2020.1.40971)
Supplement: Supplementary file 1 [file wjem-21-555-s001.docx]

**Appendix 1: Models of Wellness**

| **Model** | **Definition of Well-being** | **Components** |
| --- | --- | --- |
| Diener’s Tripartite Model of Subjective Well-being^3^ | Self-reported measure describing how people experience the quality of their life.  Involves moods, emotions and satisfaction in multiple areas of life. | **Affective balance:** frequent positive affect & infrequent negative affect involving all moods, emotions and feelings  **Life satisfaction/quality of life:** overall satisfaction as well as satisfaction in various domains such as work, relationships, financial, health, etc. |
| Ryff’s Six-factor Model of Psychological Well-being^4, 5^ | Having achieved a state of balance between challenging and rewarding life events.  Based on Aristotle’s Nicomachean Ethics. | **Self-acceptance:** positive attitude toward self  **Personal growth:** feels need to continue to develop and have new experiences over time  **Purpose in life:** believes life has meaning and has goal orientation  **Environmental mastery:** takes advantage of opportunities and feels in control of most situations  **Autonomy:** regulates behavior independent of social pressures  **Positive relations with others:** reciprocal empathy, intimacy and affection |
| Keyes’ Flourishing Model^6^ | “A state where people experience positive emotions, positive psychological functioning and positive social functioning, most of the time.”    “Living within an optimal range of human functioning, one that connotes goodness, generativity, growth, and resilience.” | High Well-being in all 3 categories without any major depression  **Emotional well-being**: positive affect, low negative affect, life satisfaction  **Psychological well-being**: self-acceptance, personal growth, purpose in life, environmental mastery, autonomy, positive relations with others.  **Social well-being:** social acceptance, social actualization, social contribution, social coherence, social integration |
| Seligman’s PERMA model^7^ | Meaningful life consists of five elements that contribute to well-being, can be pursued for their own sake, and can be defined and measured independently. | **Positive Emotions** such as happiness, joy, excitement, satisfaction, pride, awe  **Engagement** in activities that draw and build on one’s interests  **Relationships** with other people in multiple situations that produce positive emotions  **Meaning** or purpose, determining one’s “why” in every situation  **Accomplishments**: pursuit and success of mastery |
| Six Dimensions of Wellness (NWI)^8^ | “Wellness is an active process through which people become aware of, and make choices toward, a more successful existence.” | **Emotional:** self-awareness, personal identity and coping with emotions.  **Occupational:** enrichment of one’s life through work  **Physical:** physical health, mental health, nutrition  **Social:** interdependence with community and environment  **Intellectual:** development of knowledge and skills  **Spiritual:** search for meaning and purpose |
| ACEP^9^ | Based on NWI model with addition | **Emotional:** acknowledge and manage feelings, choose behavior  **Occupational:** what makes one happy to be in the ED?  **Physical:** good physical shape  **Social:** developing effective relationships  **Intellectual:** maintaining an open mind, share with others, challenge self.  **Spiritual:** meaning and purpose in emergency medicine  **Financial:** financial security |
| WELL for Life Program^10-12^ | Results of an observational and interventional study to derive 10 domains of wellness in order of importance | **Social Connectiveness:** opportunities to give and receive support  **Lifestyle Behaviors:** Especially nutrition, physicial activity, sleep; also substance use  **Physical Health:** self-perceptions of health, energy, physical sensations, physical health  **Stress & Resilience:** coping well during difficult times  **Emotional & Mental Health**  **Purpose/Meaning:** includes accomplishments and sense of why we are here  **Sense of Self:** measure of confidence and self-esteem  **Finance:** financial comfort  **Spirituality/Religiosity**  **Exploration & Creativity:** includes “pioneering” |
| Stanford Wellness Framework^13-14^  Used by the AMA | Defines physician wellness as professional fulfillment (experience happiness or meaningfulness, self-worth, self-efficacy, and satisfaction at work)* | **Culture of Wellness:** The set of behaviors, attitudes and values that promote self-care and growth (organizational responsibility)  **Efficiency of Practice:** value of clinical practice/(time and energy spent); (organizational responsibility)  **Personal Resilience:** personal skills, behaviors and attitudes that contribute to personal multi-dimensional well-being (personal obligation) |
| Mayo Clinic Engagement Model^15^ | Defines the opposite of burnout as engagement (vigor, dedication and absorption in work) | **Workload and Job Demands:** Includes specialty, team structure, compensation, and all metrics  **Efficiency and Resources:**Includes personal, team and institutional efficiency, personal organization and delegation skills, EHR  **Meaning in work:** Includes opportunities for advancement, organizational culture, personal values, physician-patient relationship  **Organizational Culture and Values:** Includes physician’s personal and professional values; organization’s mission, norms, culture  and values  **Control and Flexibility:** Includes physician personality/intentionality and organization’s degree of flexibility on a number of issues.  **Social Support and Community at Work:** Includes physician’s relationship building skills, team structure, organizational collegiality and promotion of community  **Work-life Integration:** Includes physician values and personal characteristics, organizational expectations and requirements for call and cross-coverage |
| National Academy of Medicine Conceptual Model^16^ | Clinician well-being is a multidisciplinary issue that requires a systems-thinking approach to address fully. | **Health Care Role:** Includes stage in career, patient population, all responsibilities (administrative, clinical, teaching, research, learners) and alignment of authority and responsibility  **Personal Factors:** Includes values, personality traits, social support, ability to handle stress, physical/mental/spiritual health  **Skills and Abilities:** Includes teamwork, resilience, coping skills, empathy and leadership skills  **Socio-cultural Factors:** Includes societal expectations of physicians, political and economic climate, mental health stigmatization, social determinants of health, culture of safety, implicit and explicit biases  **Regulatory, Business, and Payer Environment:** Includes compensation, documentation requirements, licensing, litigation risk, insurance company policies  **Organizational Factors:** Includes organizational culture, mission, leadership  and values; bureaucracy, diversity and inclusion, level of organizational support, professional development  **Learning and Practice Environment:** Includes autonomy, relationships, mentorship, EHR, learning, practice setting and environment. |
